# Supplementary material for: Exploring access to health and social supports for intimate partner violence (IPV) survivors during stressful life events (SLEs)—A scoping review
Source: PLoS One. 2024 Dec 2;19(12):e0313613. doi: 10.1371/journal.pone.0313613 (PMC11611170; doi:10.1371/journal.pone.0313613)
Supplement: S1 File — (PDF) [file pone.0313613.s002.pdf]

Supplement 1:

MEDLINE (OVID) Preliminary Search Strategy

1. Battered Women/
2. domestic violence/ or spouse abuse/ or gender-based violence/ or intimate partner violence/ or family violence/
3. ((wife or wives or wom#n) adj3 batter*).ti,ab,kf.
4. ((violen* or abus*) adj3 (partner* or wom#n or spous* or wife or wives or marital or marriage*)).ti,ab,kf.
5. ((domestic* or home*) adj3 (violen* or abus*)).ti,ab,kf.
6. ((relation* or interperson*) adj3 (abuse* or violen*)).ti,ab,kf.
7. (violen* adj3 (date* or dating)).ti,ab,kf.
8. (date* adj3 rape*).ti,ab,kf.
9. ((domestic* or marital or partner* or spous*) adj3 rape*).ti,ab,kf.
10. ((domestic* or marital or partner* or spous*) adj3 (sex* adj1 (abuse* or assault*))).ti,ab,kf.
11. (gender-based adj3 (violen* or abus*)).ti,ab,kf.
12. or/1-11
13. stressful life events/ disease outbreaks/ or epidemics/ or pandemics/
14. COVID-19/ or exp COVID-19 Testing/ or COVID-19 Vaccines/ or SARS-CoV-2/
15. coronavirus/ or betacoronavirus/ or coronavirus infections/
16. (disease* adj3 outbreak*).ti,ab,kf.
17. (pandemic* or epidemic*).ti,ab,kf.
18. ((new or novel or "19" or "2019" or Wuhan or Hubei or China or Chinese) adj3 (coronavirus* or corona virus* or betacoronavirus* or CoV or HCoV)).ti,ab,kf,ot.
19. (nCoV* or 2019nCoV or 19nCoV or COVID19* or COVID or SARS-COV-2 or SARSCOV-2 or SARS-COV2 or SARSCOV2 or SARS coronavirus 2 or Severe Acute Respiratory Syndrome Coronavirus 2 or Severe Acute Respiratory Syndrome Corona Virus 2).ti,ab,kf,nm,ot,ox,rx,px.
20. (longCOVID* or postCOVID* or postcoronavirus* or postSARS*).ti,ab,kf,ot.
21. (coronavirus* or corona virus* or betacoronavirus*).ti,ab,kf,ot.
22. ((Wuhan or Hubei) adj5 pneumonia).ti,ab,kf,ot.
23. Economic Recession/
24. Natural disaster/ or environmental disaster/
25. (flood* or earthquake* or tsunami* or tornado* or hurricane* or cyclone* or wildfire* or landslide* or drought* or avalanche* or heat wave* or volcan* or blizzard* or fire* or ice storm*).ti,ab,kf.
26. recession*.ti,ab,kf.
27. (econom* adj3 (depression* or uncertainty or downturn*)).ti,ab,kf.
28. or/13-26
29. health services accessibility/ or health equity/
30. social support/ or community support/ or psychosocial support systems/ or Friends/ or community
31. help-seeking behavior/
32. (health adj3 (service* or care*) adj3 (access* or inaccessib* or unreachable* or unattainab* or reach* or equit* or inequit* or use* or using or seek*)).ti,ab,kf.
33. (healthcare* adj3 (access* or inaccessib* or unreachable* or unattainab* or reach* or equit* or inequit* or use* or using or seek*)).ti,ab,kf.
34. (support* adj3 (access* or inaccessib* or unreachable* or unattainab* or reach* or equit* or inequit* or use* or using or seek*)).ti,ab,kf.ƒ

35. ((help* or assistanc*) adj3 (access* or inaccessib* or unreachable* or unattainab* or reach* or equit* or inequit* or use* or using or seek*)).ti,ab,kf.
36. ((famil* or neighbo?r* or relative* or friend*) adj3 (access* or inaccessib* or unreachable* or unattainab* or reach* or equit* or inequit* or use* or using or seek*)).ti,ab,kf.
37. (shelter* adj3 (access* or inaccessib* or unreachable* or unattainab* or reach* or equit* or inequit* or use* or using or seek*)).ti,ab,kf.
38. or/28-36
39. 12 and 27 and 37
