# Supplementary material for: Exploring access to health and social supports for intimate partner violence (IPV) survivors during stressful life events (SLEs)—A scoping review
Source: PLoS One. 2024 Dec 2;19(12):e0313613. doi: 10.1371/journal.pone.0313613 (PMC11611170; doi:10.1371/journal.pone.0313613)
Supplement: S2 File — (PDF) [file pone.0313613.s003.pdf]

Supplement 2 - Extraction Tool

**PREVIEW**<br><br>General information

Study ID

Please enter DOI/URL link it available.

Title

Title of paper / abstract / report that data are extracted from

Lead author contact details

Notes

Characteristics of included studies<br><br>Methods

Aim of study

Please enter stated aim/purpose/objectives of study.<br><br>***Key question: What is known in published literature about access to services by individuals who experience IPV during stressful life events in high-income countries?***<br>***- looking from perspectives of IPV survivors, support services providers & informal supports (family, friends, neighbours)***<br><br>Link to the protocol: https://journals.plos.org/plosone/article?id=10.1371/journal.pone.0277903

Study design

Please click on which one you think best describes study design. Ignore the Sys. Review or Text/Opinion, those were pre-populated.

1. Randomised controlled trial
2. Non-randomised experimental study
3. Cohort study
4. Cross sectional study
5. Case control study
6. Systematic review
7. Qualitative research
8. Prevalence study
9. Case series
10. Case report
11. Diagnostic test accuracy study
12. Clinical prediction rule
13. Economic evaluation
14. Text and opinion
15. Other

Start date

End date

Geography (country, region etc.)

Study funding sources

Please enter funding source and if none state "None to report"

Possible conflicts of interest for study authors

Please enter any stated conflict of interest and if none, state "None to report".

Type of Stressful Life Event

1. COVID-19/pandemic
2. Natural disaster (hurricane, flood, earthquake)
3. Economic recession
4. Other

Participants Characteristics

Participant type (check all that apply)

Please indicate if the paper is from perspective of IPV survivors or supports providers (at this point don't worry about informal/formal etc.) it will be captured in the description below.

1. Survivors of IPV
2. Service/support providers
3. Both IPV and support providers (informal or formal)
4. Other

Type of supports (formal/informal) being accessed/provided

Please indicate types of supports being accessed. Specify if formal or informal and , where possible, what the support was.

For example:
- Formal (i.e. social service providers, violence against women (VAW) services, criminal justice, health professionals, religious organizations)

-Informal (i.e., friends, relatives, neighbours)

Population description (additional information)

Is there additional information to add about sample description and size?

Information about sub-groups at higher risk (i.e., refugees, immigrants).

Sample Size of Participants

Please enter the total sample size and, if applicable, the disaggregated gender information. If possible, please add the average/mean age if reported.

	Survivors of IPV	Service Providers
total sample size		
average/mean age of sample size		
number of women		
average/mean age of women		
number of men		
average mean age of men		
Other		

Inclusion/Exclusion criteria

Please indicate any inclusion or exclusion criteria state in the study (i.e., just women, refugees, age groups, etc.). If none stated, leave blank.

Method of recruitment of participants

1. Phone
2. Mail
3. Clinic patients
4. Voluntary
5. Other

Method of data collection

1. Interviews
2. Focus groups
3. Survey/questionnaire
4. Other

Results and outcomes

Qualitative/descriptive

Key themes that emerged from qualitative analysis

Barriers

Please list barriers to accessing services during SLEs. Please indicate if there were sub-groups at higher risk.

Facilitators

Please list facilitators to accessing services during SLEs.

Lessons Learned/Recommendations

Please list any lessons learned/recommendations outlined<br><br>Quantitative

Quantitative results from the study

In this section, please indicate any key quantitative findings from the study and if significant where possible:<br><br>Examples:<br><br>- Survivors of IPV: use of IPV tool (screening, support, information) before and after if possible & significant finding; #s reached; evaluations of actual tools/approaches to provide services (if applicable).<br><br>- Service providers: 43.9% of participants (support service providers) used telephone as main remote support communication medium; % who used medium before and after stressful event (i.e., pandemic, earthquake, flood). If there was a pre/post, what the finding is and if significant.

Additional information

Please add anything else you think is pertinent and not covered in above
